# Adapting an Evidence-Based Home Visiting Intervention for Mothers With Opioid Dependence: Modified Attachment and Biobehavioral Catch-up

**DOI:** 10.3389/fpsyg.2021.675866

**Published:** 2021-08-13

**Authors:** Madelyn H. Labella, Rina D. Eiden, Caroline K. P. Roben, Mary Dozier

**Affiliations:** ^1^Department of Psychological and Brain Sciences, University of Delaware, Newark, DE, United States; ^2^Department of Psychology, Pennsylvania State University, University Park, PA, United States

**Keywords:** home visiting program, parenting intervention, parenting sensitivity, attachment, prenatal opioid exposure, neonatal opioid withdrawal syndrome (NOWS)

## Abstract

Infants born to mothers who are dependent on opioids often have difficulty regulating behavior and physiology at birth. Without sensitive maternal care, these infants are at risk for ongoing problems with self-regulation. Mothers who are dependent on opioids may experience challenges related to their substance use (e.g., unsupportive and/or risky environment, impulse control and reward system problems) that increase the likelihood of insensitive parenting in the absence of effective intervention. In this paper, we describe a home-visiting intervention we have adapted to enhance sensitive, responsive caregiving tailored to the specific needs of mothers with opioid dependence. The original intervention, Attachment and Biobehavioral Catch-up (ABC), was designed for mothers of infants aged 6–24 months who were exposed to early adversity. ABC has been shown to enhance sensitive parenting as well as children's behavioral and biological functioning, with positive outcomes extending into at least middle childhood. Mothers who are opioid dependent need earlier support than provided by ABC because opioid-exposed infants are often vulnerable at birth. The adapted intervention (modified ABC or mABC) includes one prenatal session and one early postnatal session, followed by 10 sessions every 2–3 weeks. In the initial two sessions in particular, mothers are helped to anticipate the challenges of caring for a baby who may be difficult to soothe while nonetheless providing sensitive care. mABC is intended to help mothers see the importance of responding sensitively so as to help infants overcome the developmental risks associated with opioid exposure. Additionally, mABC is structured to support mothers with the challenges of early parenting, especially if the mother herself was not parented sensitively. Throughout, the focus is on helping the mother nurture the distressed infant, attend to the infant's signals, and avoid behaving in overstimulating or intrusive ways. Case examples are presented that highlight both the challenges of working with this population as well as the gains made by mothers.

## Introduction

Opioid abuse is a public health emergency of historic proportions, affecting thousands of pregnant and parenting mothers (Clemans-Cope et al., 2019). Infants exposed to opioids prenatally are at risk for difficulty regulating physiology and behavior, particularly in the vulnerable neonatal period when many infants experience symptoms of withdrawal (Conradt et al., 2018). Sensitive caregiving is critical for helping substance-exposed infants thrive; however, mothers with opioid use disorders may struggle to parent sensitively in the context of substance-related risk factors including poverty, trauma exposure, alterations in reward processing, and emotional dysregulation. There is an urgent need for evidence-based parenting interventions to support mothers with opioid use disorders, especially in the peripartum period when infants experiencing withdrawal may be difficult to soothe. The current paper reviews existing interventions designed to enhance parenting quality among opioid-dependent mothers of infants, in addition to reviewing the evidence base of Attachment and Biobehavioral Catch-Up (ABC), a home-visiting intervention model for parents of infants exposed to early adversity. We then describe modified Attachment and Biobehavioral Catch-Up (mABC), an adaptation of ABC tailored to the needs of this population, designed to enhance parenting sensitivity and in turn promote attachment organization and self-regulation among infants with prenatal opioid exposure.

In 2017, an estimated 2.129 million American adults met criteria for opioid use disorder (Florence et al., 2021), including more than 600,000 parents living in households with their children (Clemans-Cope et al., 2019). The impact of parental opioid use begins in utero: opioids are known to cross the placenta and have been implicated in a wide range of adverse fetal outcomes (Yazdy et al., 2015). Despite these concerns, a growing proportion of American women report using opioids during pregnancy (Epstein et al., 2013; Ailes et al., 2015; Patrick et al., 2015a), with the prevalence of maternal opioid use disorder documented at delivery more than quadrupling between 1999 and 2014 (Haight et al., 2018).

The rising rate of prenatal opioid exposure is linked to a corollary increase in neonatal opioid withdrawal syndrome (NOWS), a constellation of withdrawal symptoms affecting 50–80% of opioid-exposed newborns (Patrick et al., 2015b; Conradt et al., 2019). NOWS is characterized by hyperreactivity of the central nervous system and difficulty regulating gastrointestinal, respiratory, and autonomic functions (Jones et al., 2010; Patrick et al., 2015b). Symptoms of NOWS include irritability, high-pitched cry, tremors, feeding difficulty, and disrupted sleep (Jansson et al., 2009). Even among infants without overt withdrawal symptoms, a history of prenatal opioid exposure confers risk for biological and behavioral dysregulation (Minnes et al., 2011; Nygaard et al., 2016; Reddy et al., 2017). Thus, compared to infants without prenatal opioid exposure, infants exposed to opioids in utero are at heightened risk for medical complications and broad-based difficulties with self-regulation.

Importantly, prenatal opioid use is often accompanied by exposure to other substances, both prescribed (e.g., antidepressants, sedatives) and non-prescribed (e.g., alcohol, marijuana, cocaine), many of which are known to have adverse effects on fetal development (Singer et al., 2020). For example, rates of cigarette smoking among pregnant women in treatment for opioid use disorder have been estimated at 95% (Jones et al., 2013). Prenatal opioid exposure thus occurs in the broader context of polysubstance use, with the potential for additive and/or synergistic effects on infants' outcomes (Singer et al., 2020). Co-occurring substance exposure may exacerbate risks associated with prenatal opioid use, heightening infants' vulnerability to dysregulation.

A limited body of evidence suggests that social-environmental factors, including rearing environment, may offset or moderate effects of substance-related risk on developmental outcomes (Marcus et al., 1984; Hans and Jeremy, 2001; Ornoy et al., 2001). A broader literature on risk and resilience has identified parenting as a powerful predictor of child outcomes across a wide range of adversities, including poverty and exposure to trauma (Masten and Labella, 2016). Relatedly, parenting interventions have been shown to promote resilient functioning, including healthy physiological and behavioral regulation, in the context of early adversity (Fisher et al., 2007; 2016). Taken together, the evidence suggests that sensitive parental care may be especially important for buffering effects of early vulnerability and promoting healthy development among infants prenatally exposed to opioids and other co-occurring substances (Reddy et al., 2017; Conradt et al., 2018; Finger et al., 2018).

### Challenges for Sensitive Parenting

Unfortunately, prenatal substance exposure is associated with multiple challenges that interfere with sensitive parenting. Maternal opioid use often co-occurs with poverty (Han et al., 2017; Metz et al., 2018), which in turn is associated with a range of sociodemographic risk factors (e.g., low parental education, inadequate resources) and adverse experiences (e.g., family conflict, community violence) known to undermine parenting sensitivity and healthy child development (Conger and Donnellan, 2007; Simons et al., 2016). In addition to current psychosocial stressors, pregnant and parenting women with opioid use disorders often report prior history of trauma, including physical and sexual abuse (Saia et al., 2016; American College of Obstetricians Gynecologists, 2017; Gannon et al., 2020). Early traumatic experiences confer risk for both problematic substance use and parenting dysfunction, perhaps in part by disrupting attachment processes across the lifespan (Alvarez-Monjaras et al., 2019; Labella et al., 2019; Preis et al., 2020).

The social-environmental context of maternal opioid use is thus characterized by current and historical adversity, as well as limited access to resources and social supports (American College of Obstetricians Gynecologists, 2017; Conradt et al., 2018; Peisch et al., 2018). In addition to cumulative contextual risk, women with opioid use disorders often have individual vulnerabilities that may further challenge their ability to provide sensitive care. At a physiological level, opioid dependence alters the reward system, potentially interfering with normative neurobiological processes that make parenting inherently rewarding (Kim et al., 2017; Rutherford and Mayes, 2017; Alvarez-Monjaras et al., 2019). Substance use disorders may also disrupt neural processes involved in executive function, undermining emotion regulation and impulse control (Alvarez-Monjaras et al., 2019; Peters and Soyka, 2019). The neurobiological sequelae of opioid dependence may thus impinge on skills required for effective parenting.

Relatedly, women with opioid use disorders are frequently diagnosed with a range of psychiatric comorbidities characterized by dysregulation of affect and behavior, including anxiety, depression, bipolar disorder, borderline personality, and post-traumatic stress disorder (Whiteman et al., 2014; Arnaudo et al., 2017; Preis et al., 2020). Conradt et al. (2018) propose maternal emotion dysregulation as a key vulnerability factor for psychopathology in general and substance use in particular among women using opioids during pregnancy. Maternal emotion dysregulation may function to exacerbate substance use and/or disrupt parenting directly. Difficulty regulating negative emotions may impair mothers' ability to inhibit a prepotent response (e.g., expressing anger or withdrawing from a crying infant) in order to provide sensitive care. Thus, behavioral dysregulation associated with substance use may pose additional threats to childrearing.

Prenatal opioid exposure is thus associated with a constellation of parenting risk factors at the social-environmental, behavioral, and neurobiological levels. Difficulties with sensitive parenting are multiply determined and may be compounded by infants' symptoms of NOWS. Opioid withdrawal symptoms—including fussiness, shrill cries, and disrupted sleep—may be particularly taxing for peripartum parents, contributing to frustration and fatigue (Jansson et al., 2009). Mothers of infants with severe NOWS may struggle to persist in providing comfort to an infant who is difficult to soothe. Early insensitive interactions may become entrained over time, developing transactionally into a pattern of suboptimal care that extends across infancy and beyond. Indeed, relative to community samples without prenatal opioid exposure, mothers prescribed opioid treatment medications during pregnancy interact less positively and more negatively with their opioid-exposed children during infancy, preschool age, and middle childhood (Hans et al., 1999; Salo et al., 2009; Sarfi et al., 2011). Notably, variations in observed parenting quality predict social-emotional adjustment among children prenatally exposed to opioids (Bernstein and Hans, 1994; Sarfi et al., 2013; Finger et al., 2018), illustrating the importance of sensitive parenting in this vulnerable population.

### A Review of Interventions to Promote Sensitive Care

There is an urgent need for evidence-based parenting interventions to support mothers with opioid use disorders and their infants (Peisch et al., 2018). Our review of the literature identified just four parenting programs that have been empirically evaluated with opioid-dependent mothers of infants younger than 1 year (Table 1). Two interventions (Circle of Security and patient-centered educational support groups) were tested with small pilot samples and focused primarily on feasibility and acceptability (Coleman, 2014; Kahn et al., 2017). No measures of observed parenting behavior were included, and no inferential statistics were reported, limiting evaluation of their effectiveness. A third intervention (Mothering from the Inside Out) was evaluated using a randomized clinical trial following a promising pilot (Suchman et al., 2010, 2011, 2017). This intervention was found to enhance reflective functioning among predominantly opioid-dependent mothers of infants and toddlers, although results for observed parenting behaviors were mixed (Suchman et al., 2011, 2017). Another intervention targeting mindfulness-based parenting showed promise using a pre-post design: opioid-dependent mothers of children aged 3 months through 3 years demonstrated post-intervention improvements in self-reported mindful parenting and observed parenting quality, although causal inference was limited by the lack of a comparison group (Gannon et al., 2017, 2019).

**Table 1:** Interventions targeting parenting among opioid-dependent mothers of infants.

**Intervention**	**References**	**Sample**	**Study design**	**Description of parenting findings**
Circle of Security	Coleman (2014)	8 parents enrolled in opioid treatment (children aged 6 months to 8 years)	Pre-post	Qualitative feedback indicated that the parenting program was helpful and informative.
				Self-reported caregiver reflectiveness and empowerment did not show evidence of improvement.
Educational support groups	Kahn et al. (2017)	23 pregnant and parenting women on MAT (children aged 0–5 years)	Focus groups	Qualitative feedback indicated that focus groups were perceived as helpful and supportive.
Mindfulness-Based Parenting	Gannon et al. (2017)^a^	160 pregnant and parenting women on MAT (children aged 0–3 years)	Pre-post	Observed-rated parenting quality showed large increases and self-reported mindful parenting showed small increases pre- to post-intervention.
	Gannon et al. (2019) ^a^	120 pregnant and parenting women on MAT (children aged 0–3 years)	Pre-post	Qualitative descriptions of parenting changes reported in Gannon et al. (2017) suggested increased sensitivity to child cues and supportiveness during play
	Short et al. (2017) ^a^	59 pregnant and parenting women on MAT (children aged 0–3 years)	Pre-post	Self-reported parental distress (but not parenting stress related to child difficulty or dysfunctional interactions) declined pre- to post-intervention
Mothering from the Inside Out (MIO)/Mothers and Toddlers Program (MTP)	Suchman et al. (2010)^b^	47 mothers in substance use treatment (children aged 0–3 years); predominantly opioid users (72%)	Randomized clinical pilot	At post-intervention, mothers randomized to MTP showed small advantages over mothers in the comparison group on reflective functioning and large advantages in observed parenting sensitivity; no *p*-values reported
	Suchman et al. (2011)^b^	47 mothers in substance use treatment (children aged 0–3 years); predominantly opioid users (72%)	Randomized clinical pilot	Six weeks post-intervention, mothers randomized to MTP showed medium advantages over mothers in the comparison group on reflective functioning and observed parenting sensitivity; no *p*-values reported.
	Suchman et al. (2017)	87 mothers in substance use treatment (children aged 11 months-5 years); predominantly opioid users (89%)	Randomized clinical trial	At post-intervention, the MIO group showed significantly better maternal reflective functioning (but not parent–child attachment security or observed parenting) than the comparison group
				At 3-month follow up, the MIO group showed significantly better maternal reflective functioning (but not observed parenting) than the comparison group.
				At 12-month follow-up, the MIO group showed significantly better observed parenting than the comparison group

a *Results drawn from overlapping samples*.

b *Results drawn from overlapping samples*.

*MAT, medication-assisted treatment for opioid use disorder*.

Importantly, none of these interventions are specific to early infancy. They are intended to serve a broader population of parents with children up to 3, 5, or 8 years, and as such, are not explicitly targeted to the challenges of caring for a vulnerable newborn. Additionally, only one parenting program was evaluated using a randomized clinical trial, the gold standard for assessing treatment efficacy. There remains a need for rigorous research evaluating parenting programs, particularly those that begin during the peripartum period and those that target specific parenting behaviors known to enhance self-regulation among vulnerable infants. Cioffi et al. (2019) recommend accelerating translational research aimed at supporting parents with opioid use disorder by adapting existing parenting interventions with established evidence of effectiveness in other populations.

#### A Review of Attachment and Biobehavioral Catch-Up

One promising model is Attachment and Biobehavioral Catch-Up (ABC), a ten-session home-based intervention designed to enhance parenting sensitivity, with cascading effects on parent–child attachment and child self-regulation among infants and toddlers exposed to early adversity (Dozier and Bernard, 2019). Drawing on insights from attachment theory, the ABC intervention anticipates children's innate need to seek proximity and comfort from a caregiver under conditions of threat (Bowlby, 1969/1982). Despite this need, children exposed to early adversity may have difficulty seeking out caregivers directly when they are distressed and may struggle to soothe when comfort is provided, making it more challenging for parents to provide consistent nurturing care (Stovall-McClough and Dozier, 2004). Helping young children manage distress is an important parental task, as infants rely on their parents for co-regulation of their emotions, behavior, and physiology (Hofer, 2006). Responding sensitively to children's cues when they are not distressed—and avoiding insensitive behavior that may be frightening or overwhelming—further support children's emerging self-regulation (Feldman, 2007). Sensitive parenting is especially important for infants and toddlers exposed to early adversity, who are at heightened risk for behavioral and biological dysregulation. Unfortunately, parents of vulnerable children may struggle to interact sensitively for a variety of reasons, including their own experiences of adversity, their attachment history, and/or their child's difficulty communicating emotional needs.

To support parents of vulnerable infants and toddlers in serving this crucial co-regulatory function, ABC targets three aspects of parenting behavior: (a) nurturing the child when he or she is distressed, (b) responding sensitively when the child is not distressed (i.e., following his or her lead with delight), and (c) avoiding intrusive or frightening behavior. Parent coaches deliver manualized intervention content in each of 10 weekly sessions, which are attended by parents and children. Intervention targets are illustrated using video clips of other parents and children, as well as clips of the family's interactions in previous sessions. Most importantly, parent coaches provide frequent feedback in the form of “in-the-moment” comments, which are designed to help parents recognize and engage in targeted parenting behaviors (Dozier and Bernard, 2019). In-the-moment comments have been identified as the intervention mechanism leading to changes in parenting behavior (Caron et al., 2016).

ABC was initially developed for parents and caregivers of infants aged 6–24 months and subsequently adapted for use with parents of toddlers (24–48 months). Its efficacy has been established through large RCTs with families of infant and toddlers involved in the child welfare system, as well as with families of children adopted internationally (Dozier and Bernard, 2019). Intervention effects on parenting behavior, parent–child attachment, and children's self-regulation are reviewed in Table 2. In comparison with a control intervention focused on healthy development, ABC has been shown to enhance parenting sensitivity among birth parents referred to Child Protective Services (CPS) due to concerns about child maltreatment (Yarger et al., 2016; Lind et al., 2020), as well as among foster caregivers (Bick and Dozier, 2013) and internationally adoptive parents (Yarger et al., 2020). The intervention has shown benefits for parent–child attachment in families involved with CPS: ABC has been linked to reduced attachment avoidance among infants and toddlers in foster care (Dozier et al., 2009), and to enhanced attachment security and organization among CPS-referred infants remaining in their birth parents' care (Bernard et al., 2012). When the latter sample was followed into middle childhood, children in the ABC group reported feeling more secure in their relationships with their parents than children in the comparison group, suggesting impressive longevity of effects (Zajac et al., 2020).

**Table 2:** Review of ABC effects on parenting, parent–child attachment, and child self-regulation.

**References**	**Intervention sample**	**Outcome**	**Description of findings**
**Parenting**			
Bick and Dozier (2013)	96 foster mother-infant dyads (infants aged 0–2 years)	Parenting sensitivity	At post-intervention, mothers randomized to ABC showed greater increases in sensitivity than mothers in the comparison group.
Berlin et al. (2014)	21 mothers in residential substance abuse treatment and their infants (infants aged 0–2 years)	Parenting sensitivity	At post-intervention, mothers randomized to ABC showed marginally more parenting sensitivity, consistent with a medium effect size
Yarger et al. (2016)	24 birth mother-infant dyads referred to CPS (infants aged 6 months−2 years)	Parenting sensitivity, intrusiveness	Relative to comparison mothers, mothers randomized to ABC showed greater increases in parenting sensitivity and decreases in intrusiveness across 10 sessions
Caron et al. (2016)	78 parent–infant dyads (most CPS-referred) (infants aged 0–2 years)	Parenting sensitivity, intrusiveness	Parents receiving ABC in a community setting showed increased parenting sensitivity and decreased intrusiveness from pre-to post-intervention
Roben et al. (2017)	108 parent–infant dyads (infants aged 6 months−2 years)	Parenting sensitivity	Parents receiving ABC across five community sites showed increased parenting sensitivity from pre-to post-intervention
Berlin et al. (2018)	208 low-income mother-infant dyads (most Latinx; infants aged 6–20 months)	Parenting sensitivity, intrusiveness, positive regard	At post-intervention, mothers randomized to EHS + ABC showed greater sensitivity, lower intrusiveness, and greater positive regard than mothers receiving only EHS
Lind et al. (2020)	101 birth mother-infant dyads referred to CPS (infants aged 0–2 years)	Parenting sensitivity	One month post-intervention and at a follow-up assessment 1.5 years later, mothers randomized to ABC showed more sensitivity than mothers in the comparison group
Yarger et al. (2020)	120 internationally adopted infants and toddlers and their adoptive parents (children aged 6 months−4 years)	Parenting sensitivity, intrusiveness, positive regard	Relative to comparison mothers, adoptive parents randomized to ABC showed greater increases in parenting sensitivity, decreases in intrusiveness, and increases in positive regard pre- to post-intervention. Effects persisted at a 2-year follow-up
Raby et al. (2021)	94 birth mother-infant dyads referred to CPS (infants aged 0–2 years)	Parenting sensitivity	Receiving ABC (vs. a comparison intervention) during infancy had an indirect effect on parenting sensitivity during middle childhood through parents' secure base script knowledge
**Parent–child attachment**			
Dozier et al. (2009)	46 foster mother-infant dyads (infants aged 0–3 years)	Attachment avoidance	Relative to comparison children, children whose parents received ABC showed less avoidance during distress-eliciting situations reported in a daily diary.
Bernard et al. (2012)	120 birth mother-infant dyads referred to CPS (infants aged 0–2 years)	Attachment security, disorganization	Relative to comparison children, children whose parents received ABC showed higher rates of attachment security and lower rates of disorganization in the Strange Situation
Zajac et al. (2020)	100 birth mother-infant dyads referred to CPS (infants aged 0–2 years)	Perceived attachment security	Relative to comparison children, children whose parents received ABC reported greater perceived attachment security approximately 8 years later.
**Child self-regulation: Biological**			
Bernard et al. (2015a)	100 birth mother-infant dyads referred to CPS (infants aged 0–2 years)	Diurnal cortisol	Post-intervention, children whose parents received ABC showed more normative diurnal cortisol production (higher wake-up value, steeper slope) than comparison children
Bernard et al. (2015b)	96 birth mother-infant dyads referred to CPS (infants aged 0–2 years)	Diurnal cortisol	At a three-year follow-up, preschool-aged children whose parents received ABC during infancy showed more normative diurnal cortisol production (higher wake-up value, steeper slope) than comparison children
Tabachnick et al. (2019)	96 birth mother-infant dyads referred to CPS (infants aged 0–2 years)	Respiratory sinus arrhythmia	Relative to comparison children, children whose parents received ABC showed higher respiratory sinus arrhythmia (suggesting better physiological regulation) approximately 8 years later
**Child self-regulation: Behavioral**			
Lewis-Morrarty et al. (2012)	37 foster parent–infant dyads (infants aged 0–2 years)	Executive function	Relative to comparison children, children whose parents received ABC during infancy showed better executive function as preschoolers
Lind et al. (2014)	117 birth mother-infant dyads referred to CPS (infants aged 0–2 years)	Negative affect expression	Relative to comparison children, children whose parents received ABC during infancy showed less negative affect during a frustrating task in toddlerhood
Lind et al. (2017)	121 foster parent–toddler dyads (toddlers aged 2–4 years)	Attention problems; executive function	Relative to comparison children, children whose parents received ABC in toddlerhood had fewer parent-reported attention problems and showed better executive function as preschoolers
Lind et al. (2020)	101 birth mother-infant dyads referred to CPS (infants aged 0–2 years)	Inhibitory control	Relative to comparison children, children whose parents received ABC were more likely to comply with a behavioral directive (inhibit the urge to touch forbidden toys) as preschoolers

*ABC, Attachment and Biobehavioral Catch-Up; CPS, Child Protective Services; EHS, Early Head Start*.

The benefits of ABC extend past the parenting relationship to enhance child outcomes. In particular, ABC has been found to support emerging self-regulation of biology and behavior in infants and toddlers at risk for dysregulation. Among CPS-referred families participating in a foster care diversion program, children whose parents received ABC showed more normative diurnal regulation of the hormone cortisol than children whose parents received a control intervention, an advantage that persisted in toddlerhood (Bernard et al., 2015a) and preschool (Bernard et al., 2015b). This is promising because the disruption of healthy cortisol production is believed to be one mechanism by which early stress undermines adaptive functioning and physical health across the lifespan (Gunnar and Quevedo, 2007; Fisher et al., 2016). Restoring a healthy pattern of diurnal cortisol through responsive caregiving may confer powerful protection against risks associated with early adversity (Fisher et al., 2016).

ABC's benefits for physiological regulation persist into middle childhood. In a follow-up study with the same sample, nine-year-old children whose parents received ABC during infancy showed higher respiratory sinus arrhythmia across tasks than children whose parents received a control intervention (Tabachnick et al., 2019). Respiratory sinus arrhythmia indexes parasympathetic activation, an aspect of autonomic nervous system functioning involved in maintaining homeostasis while flexibly responding to environmental demands (Beauchaine, 2001; Porges, 2007). Higher parasympathetic activation at rest is believed to reflect greater capacity for physiological and emotional regulation (Beauchaine, 2001). Thus, children in the ABC group showed enhanced self-regulation a remarkable 7–8 years after the intervention took place.

Self-regulatory benefits of ABC are also evident at the behavioral level. Relative to children in the comparison group, toddlers whose foster parents received ABC showed better self-regulation skills as preschoolers, as indexed by caregiver-reported attention problems and child performance on the Dimensional Change Card Sort, a measure of cognitive self-control skills known as executive functions (Lind et al., 2017). Furthermore, among CPS-referred families participating in a foster care diversion program, ABC was linked to less emotion dysregulation during a frustrating task in toddlerhood than seen among children in the control condition (Lind et al., 2014; Labella et al., 2020). In a follow-up study with the same sample, preschoolers whose parents received ABC were more likely to comply with a behavioral directive (i.e., not to touch forbidden toys) than those whose parents received the control intervention (Lind et al., 2020).

ABC is associated with similar positive changes in parenting when delivered by community clinicians. In community-based RCTs, parents who received ABC were more sensitive and less intrusive than parents in comparison groups (Berlin et al., 2014, 2018). Furthermore, in a sample of 108 parents seen across five community dissemination sites, parents showed large increases in sensitivity from pre- to post-intervention (*d* = 0.83), comparable to effect sizes observed in university RCTs (Roben et al., 2017). These findings build confidence that ABC is feasible and effective when delivered in the community, a prerequisite for a large-scale public health intervention.

#### ABC for Mothers in Treatment for Opioid Use Disorder

The evidence base for ABC has established its efficacy and effectiveness for families exposed to multiple types of early adversity, including CPS involvement. This is relevant for mothers with opioid use disorders, many of whom become involved with CPS if their infants test positive for opioids (including opioid treatment medications) at birth (Child Welfare Information Gateway, 2020). Prior research on ABC has included parents with substance use disorders. For example, in the RCT with families participating in foster care diversion, a subset of birth parents had been referred to CPS because of concerns about parental substance use (Bernard et al., 2015a). Furthermore, a small community-based RCT demonstrated enhanced parenting sensitivity among mothers in residential substance abuse treatment randomized to receive ABC vs. treatment as usual (Berlin et al., 2014). This provides preliminary evidence that ABC may be successfully used with parents in treatment for substance use, including opioid dependence. ABC has been successfully delivered in community settings, suggesting promise as a large-scale public health intervention, and targets areas of vulnerability for families affected by prenatal opioid exposure. ABC's focus on concrete parenting behaviors may help opioid-dependent mothers respond to their infants in nurturing and sensitive ways, with downstream benefits for parent–child attachment and children's self-regulation.

ABC thus shows promise as a treatment model for parents of infants with prenatal opioid exposure. However, ABC is designed to be delivered with older infants (6–24 months) and toddlers (24–48 months) and is not well suited to support mothers during the vulnerable peripartum period, when infants may be experiencing challenging opioid withdrawal symptoms including inconsolable crying and disrupted sleep. Mothers of infants with prenatal opioid exposure may need additional early support focused on providing nurturance to newborn infants who are difficult to soothe, as well as assistance reading infant cues to avoid overstimulation.

## Modified Attachment and Biobehavioral Catch-Up

Modified Attachment and Biobehavioral Catch-Up, or mABC, builds on the principles of ABC, tailored to address the specific needs of mothers in treatment for opioid use disorder. The first session of mABC is designed to occur during the third trimester in order to help pregnant mothers anticipate their crucial role in providing nurturance, even when their infant is difficult to soothe. A second session is intended to take place as soon as possible after birth and may be delivered in the hospital if the infant is being monitored or treated for NOWS. Following these initial sessions, the intervention proceeds with ABC targets, developmentally adapted for early infancy. In contrast to ABC, which meets weekly, mABC is intended to meet every 2–3 weeks. This extended schedule allows the parent coach to be available for postpartum support while also ensuring that parents have adequate opportunity to practice parenting behavior targets that are developmentally appropriate in older infancy.

Similar to ABC, mABC is a manualized intervention designed to be delivered in the home. Everyone who lives in the home is invited to participate in sessions, and parent coaches are encouraged to comment on parents' interactions with siblings as well as the target child. This intervention strategy is designed to help mothers practice targeted parenting behaviors in the context of their everyday lives, while navigating distractions, feedback from other family members, and the attentional demands of caring for other children. We believe this increases the likelihood that behavior change will generalize outside of intervention sessions and produce lasting benefits.

The manual provides a framework for introducing session content but is not intended to be treated as a script. Instead, parent coaches are encouraged to present material in a natural and conversational manner, soliciting mothers' input and ensuring understanding. Parent coaches simultaneously pay close attention to parent–child interactions unfolding in real time, interspersing content discussion with frequent feedback in the form of in-the-moment comments.

### In-the-Moment Commenting for mABC

Consistent with their role in ABC, in-the-moment comments are believed to be an important mechanism of parenting behavior change in mABC, drawing parents' attention to opportunities to engage in parenting targets and praising their efforts to do so. For the first several sessions, in-the-moment comments are exclusively positive, with the goal of cultivating a supportive and trusting relationship between the mother and parent coach. Parent coaches may “spotlight” positive aspects of problematic interactions in order to provide ample positive feedback while shaping mothers' behavior in the direction of parenting targets. When mothers follow their child's lead or behave in nurturing ways, parent coaches make in-the-moment comments containing at least one of the following components: (a) a specific behavior description (e.g., “She made a surprised face, and you made a face right back”), (b) the name of the relevant intervention target (“What a good example of following her lead!”), and (c) an associated developmental outcome (“You are helping her learn she has an effect on the world”). This timely feedback provides parents with concrete instantiations of the intervention targets discussed in session and emphasizes the importance of the behaviors for child outcomes. Through the parent coach commenting upon such behaviors at least once per minute, parents receive feedback on their intervention-relevant behaviors at least 60 times in an hour session.

As the intervention progresses, parent coaches introduce advanced comments designed to redirect problematic behaviors. Parent coaches may scaffold engagement in parenting targets by providing suggestions and gentle corrections. Toward the end of the intervention, parent coaches may encourage mothers to reflect on their behavior by asking, “What could you do to nurture right now?” or “Are you following or leading?” Consistent with procedures developed for standard ABC, the frequency and quality of parent coaches' comments are assessed using a 5-mins self-coding procedure reviewed during in-the-moment supervision.

Modifications to in-the-moment commenting procedures were developed to address the challenges of maintaining an adequate commenting rate when intervening with mothers of newborns. Frequent and unpredictable napping make it difficult to schedule sessions when a young infant is likely to be awake, and very young infants show fewer spontaneous behaviors, limiting opportunities to follow their leads. To ensure that mothers receive frequent positive feedback during early sessions, two categories of in-the-moment comments were added to mABC. “Pre-following” comments acknowledge approximations of following the lead behaviors in the absence of clear infant cues. For example, a parent coach may praise a mother for periodically looking down at her baby during conversation, reciprocating eye contact, and/or talking to the baby about what is happening around them. For example, a parent coach might say, “Even while you and I are talking, you keep checking in to see if he is awake and interested. You are so tuned in to his cues!” Similarly, “pre-nurturance” comments highlight approximations of nurturance—that is, gentle physical comfort in the absence of infant distress. For example, a parent coach might say, “You are rocking her so gently in your arms while she sleeps.”

As infants get older, their daytime sleep consolidates, and it becomes easier to schedule sessions when they are alert. At the same time, they begin to show more spontaneous behaviors, such as vocalizing and reaching, which serve as opportunities for following the lead. As this transition occurs, parent coaches make fewer “pre-following” and “pre-nurturance” comments, focusing as much as possible on the ABC parenting targets of nurturance, sensitivity, and delight. Parent coaches help mothers navigate the transition to more complex ways of following as infants progress developmentally: a parent might follow a 1-month old's lead by talking about what she is looking at, follow a 3-month old's lead by handing him the toy he is reaching for, and follow a 5-month old's lead by imitating her shaking a rattle.

### Session-by-Session Summary of mABC

The sequence of mABC sessions is intended to match the infant's developmental progress and the mother's level of receptivity (Table 3). Sessions that are more likely to elicit resistance are reserved for later in the intervention, when the relationship with the parent coach is well-established. If resistance does arise, parent coaches are encouraged to validate the mother's perspective, avoiding direct confrontation or minimization of the mother's beliefs. As the therapeutic relationship develops, parent coaches gently challenge developmentally inappropriate expectations and help each mother take her child's perspective. Hesitant mothers are encouraged to experiment by trying out parenting targets and seeing how their child responds. This experimentation is reinforced by frequent in-the-moment comments that praise the mother's efforts and draw attention to positive effects.

**Table 3:** Overview of mABC topic area by session.

**Session Sequence**	**Topic Area**	**Session goals**
Session m1	Providing nurturance and recognizing cues	Prenatal session: introduce mABC; emphasize the value of providing nurturance; discuss infant engagement and disengagement cues
Session m2	Providing nurturance and avoiding overstimulating behaviors	Early postnatal session: celebrate baby's birth; encourage persistence in nurturance; lay the foundation for sensitive responding to infant cues
Session 1	Providing nurturance	Reinforce the importance of nurturing the baby when distressed, even when difficult to soothe
Session 2	Providing nurturance when children do not elicit it	Encourage nurturance even when children do not provide clear cues that they need comfort
Session 3	Following the child's lead with delight (part 1)	Help the mother follow the child's lead and show delight during interactions, even when tempted to teach or set unnecessary limits
Session 4	Following the child's lead with delight (part 2)	Scaffold practice of following the lead with delight
Session 5	Attending to the child's signals and avoiding intrusive behavior	Help the mother resist the urge to engage in intrusive behavior
Session 6	Reducing frightening behavior	Discuss the drawbacks of parenting in ways that may be frightening; help parents develop alternative ways of interacting
Session 7	Recognizing voices from the past	Help the mother identify automatic ways of responding that make it difficult to provide sensitive, nurturing care
Session 8	Providing sensitive care even when you hear voices from the past	Develop strategies to “override” automatic responses in order to parent in sensitive, nurturing ways, even when it does not come naturally
Session 9	Consolidating gains	Review progress, practice behaviors still in need of improvement
Session 10	Consolidating and celebrating change	Consolidate gains, celebrate progress, and anticipate challenges ahead

The prenatal session (m1) introduces parents to the importance of nurturing their baby. Mothers are asked to reflect on how it may feel to provide comfort to a baby who is easy vs. difficult to soothe. Feelings of helplessness and frustration are normalized, and mothers are encouraged to persist in providing comfort even when their infants are unable to settle. Parent coaches ask mothers to practice providing nurturance by caring for an infant simulator (or, more simply, any doll or stuffed animal) while an audio track of infant crying is played. Although some parents may find this experience unusual, it provides hands-on practice with concrete nurturance behaviors and with the format of future mABC sessions, which include ample opportunities for in-the-moment commenting on parent–child interactions. Parent coaches provide frequent positive feedback throughout the nurturance activity, with the goal of helping mothers feel accepted, supported, and motivated for the parent coach to return. Finally, mothers are introduced to infants' engagement and disengagement cues, with the goal of helping them avoid overstimulating their vulnerable infants. They practice recognizing these cues in videos and photographs and reflect on how they may feel when their infants communicate a need to disengage.

The early postnatal session (m2) occurs as soon as possible after the infant's birth and often takes place in the hospital. This session is more flexible than most and is intended to help the parent coach connect with the mother and her infant during a potentially vulnerable time. The parent coach reinforces content introduced at the prenatal session while commenting as much as possible on the mother's observed interactions with her infant. Mothers are asked to describe a time they tried to comfort their baby and are praised for their efforts to nurture their infant, whether or not the infant was easily soothed. Mothers are also asked whether they have observed any times that their baby became overstimulated or signaled a need to disengage. The parent coach reinforces the importance of attending to infants' cues and lays the foundation for later discussion of following the child's lead by encouraging responsive interactions when the infant gives cues for engagement.

In some cases, families are unable to start the intervention prenatally—perhaps because they were not referred for services until after the infant was born, or because they gave birth before a planned prenatal session could occur. The latter scenario is not uncommon given elevated rates of preterm birth among mothers in treatment for opioid dependence (Stover and Davis, 2015). In these cases, the early postnatal session marks the parent's introduction to mABC. The parent coach should seek to communicate novel intervention content clearly without overwhelming the mother during a potentially challenging time. The primary focus should be on building a positive relationship and motivating the mother to engage in treatment. This is accomplished primarily through frequent in-the-moment comments, which have the added benefit of reinforcing session content without lengthy discussion or video review.

Following the early postnatal session, mABC continues with session content from standard ABC. Sessions one and two reinforce the importance of nurturing children when they are frightened or distressed. In session one, mothers are asked to reflect on common beliefs about parenting—for example, the idea that babies become spoiled if parents pick them up when they cry. Parent coaches validate mothers' perspectives while presenting research evidence that challenges these ideas. For example, mothers learn that babies whose parents respond quickly to their distress tend to cry less later in infancy (Bell and Ainsworth, 1972). Nurturance is described as a powerful way to build infants' trust and security in the parent–child relationship.

Parent coaches also help mothers recognize times that children's behavior make it challenging to provide nurturance. Mothers are shown video clips in which children do not clearly signal their need for comfort—infants turn away from their parent, appearing not to need them, or fuss and push them away. Mothers learn how this behavior may elicit “in-kind” responses: parents may be tempted to turn away from infants who appear not to need them (Stovall-McClough and Dozier, 2004). Parent coaches acknowledge how confusing these unclear signals can be and emphasize children's ongoing need for nurturance, even when their behavior does not elicit it. Mothers are praised for all their efforts to comfort their infants, especially when they do not directly seek nurturance or settle easily when soothed.

Sessions three and four focus on responding sensitively when the child is not distressed by following his or her lead with delight. This type of responsive interaction, which was introduced briefly in the first postnatal session, is described as a powerful way to help children learn to regulate their behavior and develop a sense of personal mastery. Mothers are shown video clips of parents following their children's lead by narrating, imitating, and/or physically assisting their play, as well as counterexamples of parents taking the lead by teaching, correcting, and setting unnecessary limits. Sessions include hands-on activities that give mothers' practice following their children's lead, even under circumstances that often tempt parents to take charge (e.g., by insisting the infant hold a baby book correctly or not allowing splashing during water play). Activities were adapted from those included in ABC to be appropriate for younger infants and include adaptations for developmental level. For example, in session four of mABC, parents are coached to follow their child's lead while exploring a play mat or engaging in water play.

Session five builds on the importance of following the child's lead to address intrusive parenting. Mothers are asked to reflect on their own childhood experiences with intrusive behavior, such as roughhousing and tickling. Parent coaches help mothers to take the perspective of their infants, who may feel overwhelmed and dysregulated despite appearing to enjoy intrusive play. Video examples are shown of parents playing with puppets in ways that are dysregulating to their infants, as well as in ways that are responsive to infant cues. Mothers are asked to play with their own children using puppets, stuffed animals, or other toys that can easily become overwhelming. Parent coaches use in-the-moment comments to support mothers in following their children's lead despite the potential to engage in intrusive behavior.

Session six extends insights from the previous session to address frightening behavior. Mothers are asked to recall experiences from childhood when they may have been frightened by caregivers and to reflect on how those experiences affected them. Parent coaches gently challenge responses that downplay or endorse frightening experiences, providing research evidence that harsh discipline tends to elicit more rather than fewer behavior problems in children over time (e.g., Lansford et al., 2005). Mothers are asked to consider times they may have frightened their own children (perhaps without meaning to) and to identify strategies that could help them avoid frightening behavior in the future. If parent coaches have observed frightening behavior during prior sessions, those observations may be discussed and/or illustrated with video examples. To avoid shaming mothers, coaches should take care to normalize parental frustration and provide ample counterexamples of times that they did not behave in frightening ways. Mothers are encouraged to minimize frightening behavior as much as possible in order to avoid sending mixed messages about the safety of the parent–child relationship, which would undermine their progress in providing nurturance and following their children's lead.

Sessions seven and eight address automatic ways of responding that arise from mothers' past experiences and challenge their current parenting. Parent coaches prepare for these sessions by identifying the parenting target the mother struggles with most. The parent coach presents a video clip of the mother showing strength in that domain, followed by a video clip illustrating a weakness. The parent coach helps the mother to reflect on past experiences, especially their childhood experiences with caregivers, that may contribute to their current difficulty. For example, a mother whose own parents responded dismissively to her childhood distress may struggle to provide nurturance to her infant. The mother's automatic style of responding is discussed in terms of “voices from the past” —for example, the mother who downplays her child's distress may be echoing her own mother's voice saying, “Get up, you're not a baby.” “Voices from the past” are described as a universal experience, and the ability to recognize one's “voices” is framed as a strength, enabling parents to make their own decisions about how to respond to their children in the present. Mothers are encouraged to identify strategies to help them “override” voices from the past, bypassing their automatic responses in order to parent in sensitive, nurturing ways.

As might be expected, sessions seven and eight often involve emotionally vulnerable discussions about the mother's caregiving history. In such cases, the parent coach must take care to respond supportively to the mother's disclosures while remaining attentive to parent–child interactions in the present moment. The primary focus remains on identifying and overriding “voices from the past” that interfere with parenting in the present, rather than processing potentially traumatic childhood experiences. Skillful in-the-moment commenting can help mothers continue to parent sensitively even while discussing painful “voices from the past.”

Finally, sessions nine and ten help mothers to consolidate gains from previous sessions. Parent coaches select activities that will help mothers celebrate progress while practicing skills that remain problematic. Mothers are asked to reflect on what they have learned and anticipate how they will apply mABC parenting targets as their children grow older. In the final session, parent coaches share video clips that illustrate mothers' progress over the course of the intervention. Mothers are given video montages highlighting moments from earlier sessions in which they engaged in the targeted parenting behaviors of nurturance, following the lead, and delight. Jointly viewing the montage provides a powerful opportunity to celebrate change and reinforce parenting targets that mothers can apply in the months and years to come.

### Putting mABC Into Practice

mABC is currently being evaluated through a RCT based at the University of Delaware, enrolling pregnant and recently postpartum mothers on medication-assisted treatment for opioid dependence. Although robust effectiveness data for mABC will not be available until the RCT concludes, clinicians serving peripartum mothers with opioid use disorder have identified an urgent need for appropriate parenting services. To address this need while contributing to mABC's developing evidence base, community partners in Maine and a growing number of other dissemination sites have begun to implement mABC and evaluate its effectiveness using a pre-post design. mABC is being implemented in Maine through a hospital-based healthcare system at MaineGeneral Medical Center, with frequent supervision and consultation provided by the University of Delaware. Funding from the John T. Gorman Foundation supported the training and time of two local parent coaches, who recruited mothers dependent on opioids through family practice obstetrics offices. The parent coaches in Maine—the first to train in mABC outside of the RCT—have been crucial in further refining communication strategies with mothers in the perinatal period, identifying challenges in recruitment and retention, and creating supervision and dissemination tools for pre-nurturance and pre-following comments. mABC is currently being implemented in multiple states and settings, with early fidelity and parental sensitivity data supporting community effectiveness.

Because of the unexpected challenges of a global pandemic, mABC is being delivered through telehealth in addition to home visiting. Transitioning mABC to telehealth has felt remarkably successful. To date, most mothers have had access to Internet-connected devices (primarily smartphones, but also computers and tablets), which are used to videoconference with their parent coaches. In the RCT, a minority of mothers have needed assistance obtaining appropriate devices. The research team has purchased two WiFi-enabled tablets and two smartphones with prepaid data plans for participating mothers to use during telehealth sessions; these devices cost approximately $50 each, and the data plans cost $35–$45 monthly for the duration of the intervention. Mothers are also supplied with inexpensive phone stands that allow them to prop up phones or tablets during sessions. They are encouraged to set up their devices so both they and their children are visible on screen, facilitating in-the-moment commenting. Given that parent coaches' in-the-moment comments are key to intervention fidelity and to effectively engaging the intervention mechanism of parental sensitivity, making such comments was critical to success. We have found through data collected in our dissemination sites that parent coaches maintain high rates of in-the-moment comments when implementing ABC through telehealth (Roben et al., 2021).

## mABC Case Examples

### Emily

Emily was a 35-year-old single mother pregnant for the fifth time when she enrolled in mABC. Her two oldest children had been born while she was in the midst of active addiction to prescription opioids and her parental rights with these children had been terminated. Emily then enrolled in a methadone maintenance program and began abstaining from illicit opioid use. When she enrolled in mABC, Emily had been taking methadone for 3 years, during which time she had given birth to Ben (age two) and Grace (age one). She had not intended to become pregnant again so quickly and felt overwhelmed at the prospect of having 3 children under 3 years. Familiar with reporting rules from previous pregnancies, Emily was anxious about coming to the attention of CPS if her baby tested positive for methadone at birth.

At the prenatal session, Emily appeared insecure in her parenting. She seemed anxious to impress the parent coach, encouraging her son to show off his counting skills, repeatedly correcting his play (“No, not like that, you know how to hold the book!”). Emily apologized to the parent coach when her daughter cried (“I don't why she's so fussy, she's never like this! She only ever cries when she's hungry!”) before shushing her and trying to distract her with a bottle of juice. The parent coach capitalized on a brief moment of nurturance, when Grace rested her head on her mother's lap and Emily briefly touched her back. “Look at that—even more than the juice, your gentle touch is helping her feel better! That is exactly what we're going to talk about today—how important it is to show your children nurturance when they are upset.” Emily lit up. When the parent coach encouraged her to practice soothing the infant simulator, Emily pulled Grace close with one arm while she rocked the doll in the other. The parent coach praised her for gently “comforting” the crying infant simulator and added a pre-nurturing comment about Grace: “Great job sticking with it—you just kept rocking the baby even she didn't settle right away. And at the same time, you cuddled Grace close you—I can tell, being next to mom is her favorite place in the world!”

When her new baby Evie was born, Emily invited the parent coach to meet them at the hospital. She whispered to the parent coach that hospital staff had alerted CPS when the infant tested positive for both methadone and marijuana. She would be permitted to take the baby home from the hospital if she followed a plan of safe care developed with a CPS caseworker and remained consistent with substance use treatment. During the postnatal session, Emily nervously deferred to the NICU nurses, especially after one criticized her for letting Evie fall asleep before she finished her bottle. Emily was hesitant to pick Evie up after feeding, not wanting to disrupt the tangle of monitor wires. With the encouragement of the parent coach, however, she picked Evie up when she fussed and rocked her gently in her arms. The parent coach praised Emily for giving Evie the nurturance she needed from her mother, even while her medical needs were met by hospital staff. Emily gazed down at her baby proudly.

Evie was hospitalized for the next 6 weeks while she was treated for opioid withdrawal. Emily felt worried and guilty. Her older children, although also exposed to methadone, had less severe symptoms of NOWS than Evie and were home within 2 weeks. During the hospitalization, Emily struggled to balance Evie's needs with the needs of her young children at home. The parent coach checked in supportively by phone, reminding Emily that even though she could not be with all her children all the time, she was letting them know she was there for them every time she comforted them.

When Evie was discharged, mABC sessions resumed at home with all three children present. Emily responded well to in-the-moment comments focused on following her children's lead and showing delight. She became less inclined to correct and teach, instead narrating her children's play and imitating Evie's cooing and babbling. Encouraged by pre-following comments, she moved toys closer to Evie's reach, rather than putting them directly in Evie's fist. Despite her initial progress, Emily continued to struggle with nurturance. Evie had more difficulty soothing than her older siblings had, which Emily attributed to her more severe NOWS symptoms. “I just feel like the worst mom, because it's my fault,” she told her parent coach. Overwhelmed by Evie's crying, Emily would go down a list of potential problems to fix—offering a bottle, giving baby Tylenol, changing and re-changing her diaper. With the older children, Emily was more irritable, tersely telling them to stop crying— “I just fed you, you're okay.” With scaffolding from her parent coach, Emily was able to show nurturance, but providing comfort was rarely her first reaction.

During session seven, the parent coach showed Emily video clips of times that she offered nurturance right away, as well as times that she was slow to comfort, focusing instead on problem-solving. She drew Emily's attention to Evie turning around to reach for her mother while Emily distractedly hunted for a pacifier, missing her baby's bid for physical comfort. With her parent coach's help, Emily identified the automatic thought, “My children need something else—I am not enough.” She connected this thought to feelings of shame about her opioid use: Emily blamed herself for their symptoms of NOWS, a message that was reinforced by family members who saw her as a drug addict and judged her parenting. With her parent coach's help, Emily began the process of “overriding” this automatic thought, telling herself, “I know my children need me.” For the remaining sessions, Emily worked with her parent coach on “nurturing first” —picking up her children and asking them gently if they were okay before offering other solutions. She was astonished how often they settled without needing anything more.

### Monique

Monique was 8 months pregnant with her second child when she enrolled in mABC. She lived with her older sister Frances, who had legal custody of Monique's 3-year-old daughter Amaya due to ongoing concerns about parental substance use. Monique had been actively using heroin when she discovered she was pregnant again at 4 months' gestation. At Frances's encouragement, she sought out medication-assisted treatment and temporarily moved into a sober living home, where the prenatal session took place. As the session began, Monique appeared skeptical and closed off, responding monosyllabically as the parent coach sought to engage her in conversation about comforting an infant who is difficult to soothe. When encouraged to practice nurturance with the infant simulator, Monique was initially awkward, stiffly holding the doll on her lap. As the parent coach praised her efforts with a series of in-the-moment comments, Monique appeared to soften, smiling and gently jiggling the baby in her arms. She shifted the infant simulator to a more comfortable position against her chest, commenting, “This is how my daughter always liked to be held.” The parent coach praised her, “You play such an important role in helping Amaya settle down when she's upset. You'll do the same thing for your new baby, just like you are doing with this pretend one! He's crying and crying, and you just keep gently rocking him, smoothly moving him to a more comfortable position. That's really going to let him know you're there for him - you can stick with it when he is upset.”

Monique gave birth to baby Elijah a few weeks later. After being treated briefly for mild NOWS, he was discharged home. Because children were not allowed in the sober living facility, Monique and Elijah moved back in with Frances and Amaya, and mABC sessions resumed in the home. Frances declined the parent coach's invitation to join the intervention but was often present for several minutes at the beginning or end of sessions. During the first few sessions, Monique appeared exhausted and emotionally flat, rarely smiling or interacting spontaneously with baby Elijah, who lay listlessly on her lap. She was visibly overwhelmed by Amaya, an energetic child who became easily dysregulated when her mother's attention was focused elsewhere. During the first session, Amaya picked up her aunt's embroidered cushions and threw them across the room. Monique grabbed her arms roughly and yelled sharply at her— “You know auntie don't let you touch those!” Amaya yelled and kicked, tears welling up in her eyes. The parent coach commented, “This is really challenging—you know Amaya is upset but it's so hard to provide comfort when she's pushing you away.” When Amaya had an outburst several minutes later, Monique sighed deeply. The parent coach said gently, “It would be so easy to yell at a time like this, but you're working really hard to stay calm.” Monique looked at the parent coach gratefully and replied, “I'm so tired. Elijah barely slept at all last night.” The parent coach built on this moment of connection using a pre-nurturance comment: “That makes staying calm even more impressive. And even though you're totally exhausted, you're holding Elijah so gently in your arms—look how comfortable he looks nestled against you!” Monique looked down at Elijah with a hint of a smile. The parent coach immediately commented on this flicker of delight, “Aww, look at you smiling down at him. That's going to let him know how much you love and enjoy him.”

The next few sessions proceeded in a similar way. When the parent coach arrived, Elijah was often lying passively in his car seat. With encouragement, Monique would pick him up and hold him, but she often appeared preoccupied with Amaya's behavior. When Amaya became upset and acted out, Monique responded with frustration that bordered on being frightening. The parent coach framed these difficult moments as examples of “unclear cues” —when a child needs comfort but has difficulty seeking it directly. Monique's sister Frances, passing through the living room, expressed skepticism: “Amaya knows exactly what's she's doing.” The parent coach did not directly confront this resistance, responding, “It can be hard to tell! And sometimes what they need most is just to know that a parent is there for them when they're upset. I know it's so challenging, but what if you experimented with offering comfort when Amaya seems frustrated?” Frances snorted but Monique agreed to try.

Monique's initial efforts were awkward and stilted, but she persisted with encouragement from her parent coach. Gradually the tone of her responses to her children changed. Monique became far more likely to respond with physical comfort, rubbing Amaya's back or picking her up when she started to cry and yell. Instead of acting out, Amaya began to seek out comfort by climbing on Monique's lap and cuddling with her mother and brother. The parent coach took care to praise Monique's parenting of both children: “Look at that, you were holding Elijah and Amaya wanted to join in. Now you're holding them both at the same time—it's not easy to meet everyone's needs at once, but you're doing it!”

After the introduction of following the lead, Monique became more animated and interactive with both children. She had a strong tendency to take the lead during play—for example, shaking Elijah's arm while he held a rattle, and instructing Amaya how to build with blocks. With scaffolding from her parent coach, Monique was able to observe that behaving intrusively upset Elijah and worsened Amaya's behavior. Over time, she adjusted her approach. She began following their leads—saying “yum, yum, yum” when Elijah put toys in his mouth and joining in when Amaya sang Baby Shark. As their interactions became easier, Monique smiled and laughed more readily, showing her children she delighted in them. She looked like a different person from the exhausted, frustrated parent at the first postnatal session.

Frightening behavior still emerged from time to time, usually directed at Amaya. Monique was able to see that yelling and threatening escalated tense interactions with Amaya and caused Elijah to startle. While discussing voices from the past, Monique recalled that her mother pushed her to be “tough,” often yelling at Monique to stop crying and spanking to enforce rules. Monique described becoming less open, hiding her feelings and concealing problems from her mother so she wouldn't get in trouble. Monique realized that she did not want the same thing for her own children—she wanted them to feel safe and secure in her love for them. Remembering her own early entry into substance use, Monique said she wanted her children to feel comfortable coming to her with problems so she could help. The parent coach praised these insights and highlighted Monique's progress: “You've been working so hard to show them you're always there for them when they're upset. You want them to know you're always a safe person to come to.” Monique agreed that she did not want frightening behavior to send a mixed message to her children and committed to working on overriding her frustration by saying “I want my kids to know they're safe and loved.” Monique continued to make progress and consolidate gains during her final few sessions. Although at times she was slightly intrusive or spoke with annoyance, she worked hard to stay calm, offer comfort, and follow her children's lead. Monique became emotional watching the video montage presented in the final session. Turning to the parent coach, she said, “I don't always feel like I do enough as a mom. This makes me feel like enough.”

## Conclusion

Modified Attachment and Biobehavioral Catch-Up, or mABC, was designed to address limitations in prior intervention research and meet the needs of mothers and infants affected by prenatal opioid exposure. mABC is adapted from Attachment and Biobehavioral Catch-Up, a home-visiting intervention shown to enhance parenting sensitivity, parent–child attachment, and children's self-regulation among families of infants and toddlers affected by early adversity. As such, mABC has a strong theoretical and empirical foundation. Consistent with the original intervention, mABC targets areas of potential vulnerability for mothers with opioid use disorder—namely, nurturing an infant who is difficult to soothe, responding sensitively by following the child's lead with delight, and avoiding intrusive or frightening behavior. As with ABC, mABC is targeted in focus, tailored to the individual, and supportive in tone. To address the specific needs of peripartum mothers with opioid use disorders, mABC is designed to begin prenatally or shortly after birth, with an expanded emphasis on soothing a fussy newborn and avoiding overstimulation. This adapted intervention is currently being implemented in the context of a university RCT and community practice. Flexible implementation via telehealth amid the COVID-19 pandemic represents an additional strength of this approach.

Because research is ongoing, there is not yet published data directly supporting the effectiveness of mABC, which is a limitation of this review. Important future directions include establishing evidence of the efficacy and effectiveness of mABC in both university and community settings. We anticipate direct effects of mABC on parenting sensitivity, with downstream benefits for children's self-regulation at both biological and behavioral levels. Once such evidence has been established, future research may fruitfully evaluate the impact of delivery method (i.e., in-person vs. telehealth vs. hybrid delivery) and identify moderators of treatment effectiveness in order to tailor therapeutic approach for the needs of individual families. Additionally, mABC may be tested among other populations at risk for early parenting difficulties and child dysregulation. For example, families affected by perinatal depression, parental emotion dysregulation, and/or premature birth may benefit from mABC's focus on sensitive parenting during early infancy in the context of risks to healthy self-regulatory development.

Much more research is needed to inform and evaluate parenting interventions designed for families affected by prenatal opioid exposure. Drawing from decades of research demonstrating the effectiveness of Attachment and Biobehavioral Catch-up, modified ABC shows strong promise for enhancing parenting sensitivity and children's self-regulation in families affected by maternal opioid use.

## Author Contributions

MD developed the mABC intervention in collaboration with RE and CR. All authors contributed to the conception of the manuscript. ML wrote the first draft of the manuscript. All authors contributed to manuscript revision and approved the submitted version.

## Conflict of Interest

The authors declare that the research was conducted in the absence of any commercial or financial relationships that could be construed as a potential conflict of interest.

## Publisher's Note

All claims expressed in this article are solely those of the authors and do not necessarily represent those of their affiliated organizations, or those of the publisher, the editors and the reviewers. Any product that may be evaluated in this article, or claim that may be made by its manufacturer, is not guaranteed or endorsed by the publisher.
